# Titanium Surface Coating with a Laminin-Derived Functional Peptide Promotes Bone Cell Adhesion

**DOI:** 10.1155/2013/638348

**Published:** 2013-03-25

**Authors:** Seung-Ki Min, Hyun Ki Kang, Da Hyun Jang, Sung Youn Jung, O. Bok Kim, Byung-Moo Min, In-Sung Yeo

**Affiliations:** ^1^Department of Oral and Maxillofacial Surgery, School of Dentistry, Seoul National University, Seoul 110-749, Republic of Korea; ^2^Department of Oral Biochemistry, School of Dentistry and Dental Research Institute, Seoul National University, Seoul 110-749, Republic of Korea; ^3^Department of Prosthodontics, School of Dentistry and Dental Research Institute, Seoul National University, Seoul 110-749, Republic of Korea

## Abstract

Laminin-derived peptide coatings can enhance epithelial cell adhesion to implants, and the positive effect of these peptides on bone cell adhesion has been anticipated. The purpose of this study was to evaluate the improvement in bone cell attachment to and activity on titanium (Ti) scaffolds coated with a laminin-derived functional peptide, Ln2-P3 (the DLTIDDSYWYRI motif). Four Ti disc surfaces were prepared, and a human osteosarcoma (HOS) cell attachment test was performed to select two candidate surfaces for peptide coating. These two candidates were then coated with Ln2-P3 peptide, a scrambled peptide, or left uncoated to measure cell attachment to each surface, following which one surface was chosen to assess alkaline phosphatase (ALP) activity and osteogenic marker gene expression with quantitative real-time PCR. On the commercially pure Ti surface, the Ln2-P3 coating significantly increased cellular ALP activity and the expression levels of ALP and bone sialoprotein mRNA as compared with the scrambled peptide-coated and uncoated surfaces. In conclusion, although further in vivo studies are needed, the findings of this in vitro study indicate that the Ln2-P3-coated implant surface promotes bone cell adhesion, which has clinical implications for reducing the overall treatment time of dental implant therapy.

## 1. Introduction

Short functional peptides have been reported to induce numerous cellular activities without immune rejection [1]. Indeed, several synthetic peptides derived from the five carboxyl-terminal large globular (LG) domains of the laminin *α*2 chain promote cell adhesion [2–5]. Since the surfaces of implants designed for bone repair strategies are often modified to enhance cell adhesion between the host bone and the implant surface [6–9], laminin-derived peptide-coated implant surfaces are also anticipated to show stronger bone responses than uncoated surfaces. Recently, the DLTIDDSYWYRI motif (amino acids 2221–2232; Ln2-P3) from the human laminin *α*2 LG domain was reported to play a role in cell adhesion through syndecan-1 and the protein kinase C*δ* signaling pathway [2]. While it is known to be involved in adhesion across a broad range of cell types [2], Ln2-P3 has been chiefly evaluated in terms of its effectiveness in promoting epithelial cell attachment rather than in estimating its affinity for bone cells [10–12]. A few studies have been found to test the bone response of a laminin-coated titanium (Ti) surface [13–16]. However, the coated material has been laminin, the protein itself, not a peptide derived from it.

The aim of this study was to investigate the attachment and osteoblastic gene expression of osteoblast-like cells seeded onto an Ln2-P3-coated Ti implant surface in vitro using the human osteosarcoma (HOS) cell line. The hypothesis underlying this study is that Ln2-P3 would help improve the biocompatibility of the implant surface and facilitate bone cell attachment.

## 2. Materials and Methods

### 2.1. Preparation and Characterization of the Ti Discs

Ti discs (20 mm in diameter, 0.5 mm thick) were prepared from commercially pure (c.p.), grade 4 Ti. Four disc surfaces were prepared to determine an appropriate surface for peptide application. A c.p. Ti surface, without any surface modification, served as a control. The second Ti surface was sandblasted with large grit and acid etched (SLA surface; Dentium Co. Ltd., Suwon, Korea) [17, 18]. The third Ti surface was anodized, as described in a previous study (Dentium Co. Ltd., Suwon, Korea) [17], and the fourth Ti surface was coated with calcium phosphorus (Ca-P), also described previously [17]. The chemical composition and roughness of each surface was analyzed with an electron probe microanalyzer (EPMA; JXA-8900R, Jeol Ltd., Tokyo, Japan) and confocal laser scanning microscope (CLSM; LSM 5-Pascal, Carl Zeiss AG, Oberkochen, Germany), respectively.

### 2.2. Cells and Peptides

Human osteosarcoma (HOS) osteoblast-like cells were purchased from the American Type Culture Collection (ATCC, Rockville, MD, USA) and cultured in Dulbecco's modified Eagle's medium (DMEM, Gibco BRL, Carlsbad, CA, USA) supplemented with 10% fetal bovine serum (FBS). Peptides (scrambled peptide or Ln2-P3 peptide) were synthesized by the Fmoc (9-fluorenylmethoxycarbonyl)-based solid-phase method with a C-terminal amide using a Pioneer Peptide Synthesizer (Applied Biosystems, Foster City, CA, USA) and were purified and characterized at Peptron (Daejeon, Korea). The purity of all peptides used in this study was more than 95%, as determined by high-performance liquid chromatography.

### 2.3. Attachment Assay for Candidate Surface Selection

A total of 12 Ti discs (three discs for each surface, 20 mm in diameter, 0.5 mm thick) were placed into the wells of 12-well culture plates. Monolayers of routinely cultured HOS cells were detached by trypsin digestion, and 1 mL of a cell suspension containing 2 × 10^5^ HOS cells was pipetted onto the disc surface in each well. The cells were allowed to settle/adhere for 1 h at 37°C in a 5%  CO_2_ atmosphere. Loosely adherent or unbound cells were removed by aspiration, and the wells were washed once with PBS. The remaining bound cells were fixed with 10% formalin in PBS for 15 min and stained with 0.5% crystal violet for 1 h. The wells were gently rinsed with double-distilled water three times and lysed with 2% SDS for 5 min. Absorbance was measured at 570 nm in a model 550 microplate reader (Bio-Rad, Hercules, CA, USA).

### 2.4. Cell Attachment Assay

The two surfaces with the strongest bone responses determined by the crystal violet assay were then selected for further analysis. A total of 24 discs (20 mm in diameter, 0.5 mm thick) for each surface were coated with either a scrambled peptide (SP, 23 *μ*g/cm^2^) or Ln2-P3 (23 *μ*g/cm^2^) or left uncoated, with eight discs per group. These discs were then placed into the wells of 12-well plates, washed once with PBS, and seeded with 1 mL of a cell suspension containing 1 × 10^5^ HOS cells. The cultures were incubated for 1 h and 24 h (1 day) at 37°C in 5%  CO_2_. The loosely adherent or unbound cells from the experimental wells were removed by aspiration, the wells were washed once with PBS, and the remaining bound cells were fixed in 4% paraformaldehyde in PBS for 15 min. The fixative was aspirated. After washing in the buffer, the Ti plates were dehydrated in a graded series of ethanol solutions. After critical point drying (HCP-2, Hitachi, Tokyo, Japan), the samples were sputtered with Au/Pd using an SEM coating system (Quorum Q150T-S, Quorum Technologies Ltd, West Sussex, UK), and a field emission scanning electron microscope (FE-SEM; Hitachi S-4700, Hitachi, Tokyo, Japan) at 15 kV was used to determine the HOS cell attachment ratios between the Ti surfaces and the various coatings. To ensure a representative count, each Ti disc was divided into quarters, and one field per each quarter was photographed. A comparison between the cell attachment levels on the peptide-coated surfaces was used to choose a final Ti surface to test for changes in alkaline phosphatase (ALP) activity and the expression of osteogenic markers using quantitative real-time PCR.

### 2.5. Alkaline Phosphatase Activity Assay

In 60 mm culture dishes, the selected Ti discs (50 mm in diameter, 0.5 mm thick) were coated with SP or Ln2-P3 (23 *μ*g/cm^2^) by drying for 18 h at room temperature and then washed once with PBS. Monolayers of HOS cells from routine culture were detached by trypsin digestion, and 3 mL of a cell suspension containing 8 × 10^5^ cells was placed onto each Ti surface or into the wells of the control plastic dishes. The cells were cultured for 1 day or 3 days at 37°C in a 5%  CO_2_ atmosphere. The medium was changed every 2 days. At the end of the incubation, Ti discs were transferred to new 60 mm culture dishes. ALP activity was assayed in a reaction mixture composed of 8 mM *p*-nitrophenyl phosphate (Calbiochem, San Diego, CA, USA), 0.1 M glycine-NaOH buffer, pH 10.4, 150 mM MgCl_2_, 150 mM ZnCl_2_, and 15 *μ*L of cytosol extract in a final reaction volume of 90 *μ*L. The reaction was incubated for 1 h in a water bath at 37°C and terminated by the addition of 210 *μ*L of 0.25 M NaOH. The absorbance was measured at 405 nm in a Bio-Rad Model 550 microplate reader (Bio-Rad). Enzyme activity was expressed as nmol of *p*-nitrophenol product per min per *μ*g of protein.

### 2.6. Quantitative Real-Time RT-PCR

Total RNA was isolated using the RNeasy Mini Kit (Qiagen, Valencia, CA, USA), according to the manufacturer's instructions. RNA was denatured by 70°C incubation for 10 min and kept on ice for 5 min. cDNA was prepared using SuperScript III Reverse Transcriptase (Invitrogen, Carlsbad, CA, USA) and a random hexamer (Fermentas, Hanover, MD, USA) and then subjected to real-time PCR amplification using SYBR Green PCR Master Mix (Takara, Shiga, Japan) containing a 300 nM final concentration of each primer and cDNA corresponding to 17 ng of total RNA. Real-time PCR was performed using the 7300 Real-Time PCR System (Applied Biosystems, Foster City, CA, USA). Primer sequences were designed using Primer Express Software version 3.0 (Applied Biosystems, Foster City, CA, USA). The PCR primers used were as follows: ALP, 5′-CCCACGTCGATTGCATCTCT-3′ (sense) and 5′-AGTAAGGCAGGTGCCAATGG-3′ (antisense); bone sialoprotein, 5′-AAGGCTACGATGGCTATGATGGT-3′ (sense) and 5′-AATGGTAGCCGGATGCAAAG-3′ (antisense). After incubation at 95°C for 4 min, PCR cycling conditions consisted of 40 cycles at 95°C for 15 sec, 60°C for 20 sec, and 72°C for 34 sec. To analyze the data, cycle threshold values were determined by automated threshold analysis with Sequence Detection Software version 1.4, after which the calculated cycle threshold values were exported to Microsoft Excel for analysis. The relative expression of each target mRNA was calculated using the comparative cycle threshold method according to the manufacturer's procedures (Applied Biosystems, Foster City, CA, USA). 

### 2.7. Statistical Analyses

Statistical analysis of the data was performed with R software (version 2.12.0, R Foundation for Statistical Computing, Vienna, Austria). The results were compared by an analysis of variance (ANOVA). When significant differences were found, Scheffe's post hoc analysis was used. *P* values less than 0.05 were considered significant.

## 3. Results

**Figure 1:**
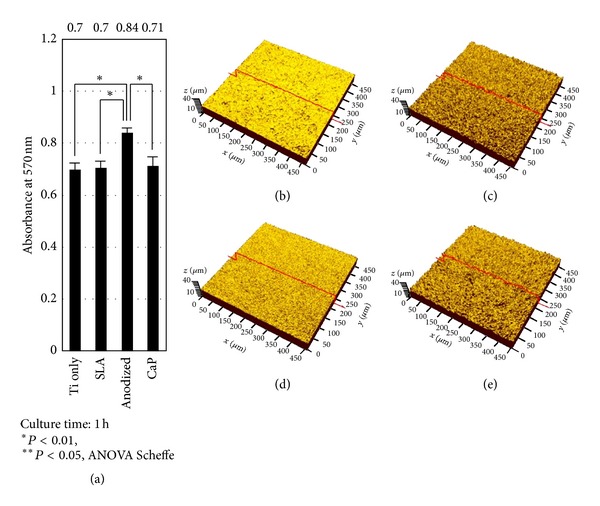
Crystal violet assay for the determination of HOS cell attachment and confocal laser scanning microscopy (CLSM) for the assessment of surface roughness. (a) Using a crystal violet cell attachment assay, the anodized surface showed the highest cell attachment (as indicated by the highest absorbance), with no significant differences among the other surfaces. (b–e) The morphologies of the commercially pure (c.p.) titanium (Ti) (b), sandblasted with large grit and acid-etched (SLA) Ti (c), anodized Ti (d), and calcium phosphorous- (Ca-P-) coated Ti (e) surfaces are shown. Three-dimensional (3D) roughness parameters were measured from the CLSM images. *S*_a_ is defined as the arithmetic average of the 3D roughness, representing the average height deviations of a given surface area. S_dr_ is defined as a developed area ratio, representing the extent of surface enlargement if a given surface is flattened. Red lines are the cross-sectional lines where 2D roughness parameters were measured. Data are expressed as the mean ± standard deviation (*n* = 3). Ti only: c.p. Ti.

The EPMA and CLSM analyses indicated that the chemical composition and roughness of each surface were similar to those reported in previous studies (Figure 1 and Table 1) [19–22]. Figure 1 shows the absorbance results from the crystal violet cell attachment assay. The anodized Ti surface had a significantly higher cell attachment than any of the other investigated surfaces (*P* < 0.01). There were no significant differences among the c.p. Ti, SLA-treated and Ca-P-coated surfaces. Because the c.p. Ti surface offers the advantage that additional modification procedures are unnecessary, the anodized and c.p. Ti surfaces were selected for peptide application.

**Figure 2:**
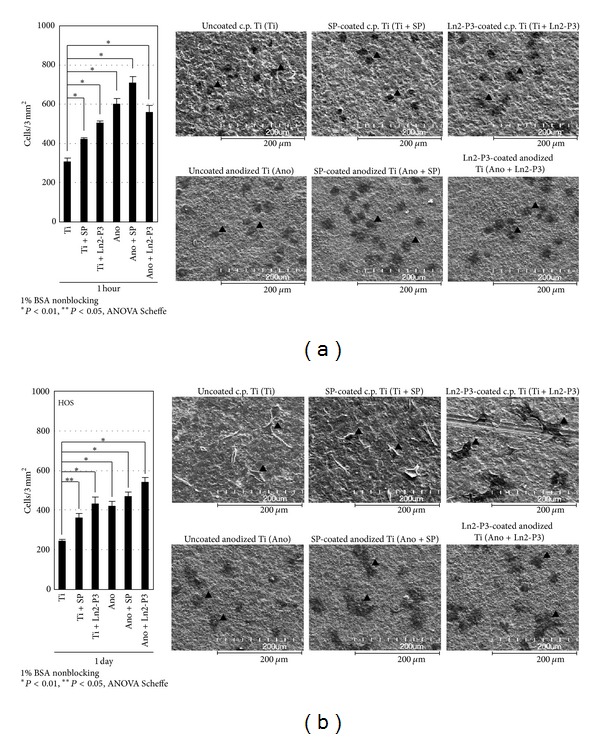
Cell attachment and FE-SEM images for commercially pure (c.p.) titanium (Ti) or anodized Ti. The dark grey area represents the attached HOS cells (black arrows). Although spreading of the attached cells was not measured in this study, cell spreading on the c.p. Ti- or anodized Ti-coated surfaces was similar, irrespective of the base surface. Ti, Ti + SP, and Ti + Ln2-P3 on the graphs mean the uncoated, SP-coated, and Ln2-P3-coated c.p. Ti surfaces, respectively. Also, Ano, Ano + SP, and Ano + Ln2-P3 mean the uncoated, SP-coated, and Ln2-P3-coated anodized Ti surfaces. Data are expressed as the mean ± standard deviation (*n* = 4). (a) The counted number of the attached cells after 1 h of culture was 307 ± 17, 421 ± 7, and 506 ± 10 for the Ti, Ti + SP, and Ti + Ln2-P3 groups, respectively. The number for the anodized Ti surfaces was 602 ± 28 (Ano), 711 ± 32 (Ano + SP), and 560 ± 33 (Ano + Ln2-P3). (b) The counted number of the attached cells after 24 h of culture was 243 ± 7, 362 ± 22, and 431 ± 35 for the Ti, Ti + SP, and Ti + Ln2-P3 groups, respectively. The number for the anodized Ti surfaces was 419 ± 25 (Ano), 469 ± 22 (Ano + SP), and 540 ± 25 (Ano + Ln2-P3).

Figure 2 shows the cell attachment assay results when the HOS cells were applied on the c.p. and anodized Ti surfaces with/without peptide coating. Without coating, the anodized surface still retained a significantly higher number of attached cells than the c.p. surface after 1 h and 1 day of culture (*P* < 0.01). However, after Ln2-P3-coating, there was no significant difference in the number of attached HOS cells to the c.p. or the anodized Ti surfaces after either 1 h or 1 day of culture (*P* > 0.05). The c.p. Ti surface was, therefore, finally determined to act as a base surface to analyze alkaline phosphatase activity and the mRNA expression of bone markers.

**Figure 3:**
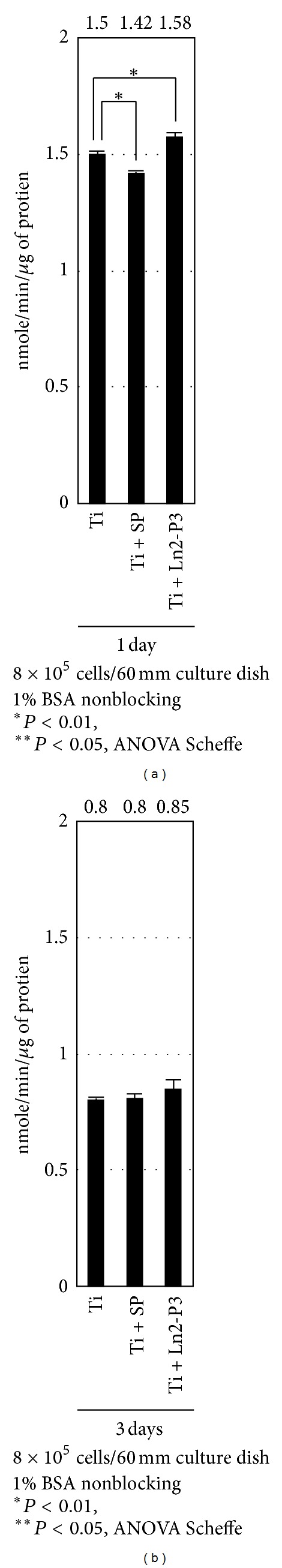
Alkaline phosphatase assay. (a) Alkaline phosphatase (ALP) enzyme activity in HOS cells seeded onto the Ln2-P3-treated titanium (Ti) discs was significantly increased as compared with that of the uncoated and SP-coated discs after 1 day. Note that the enzyme activity on the SP-coated surface was significantly lower at day 1 than that on the commercially pure (c.p.) Ti surface with no coating. (b) No significant differences in the ALP activity were observed after 3 days in culture. Data are expressed as the mean ± standard deviation (*n* = 3). Ti, Ti + SP, and Ti + Ln2-P3 on the graphs mean the uncoated, SP-coated, and Ln2-P3-coated c.p. Ti surfaces, respectively.

**Figure 4:**
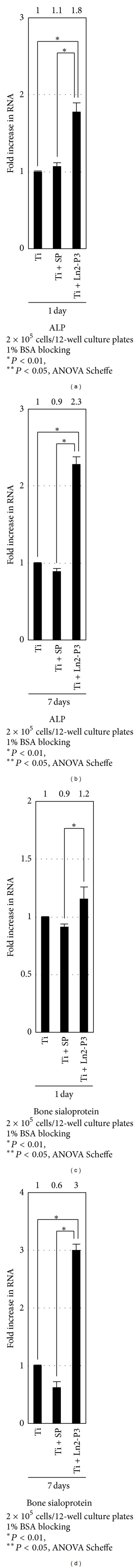
Osteogenic gene expression profiles. Induction of the ALP (a and b) and bone sialoprotein (c and d) gene expression on the uncoated, SP-coated, and Ln2-P3-coated commercially pure (c.p.) titanium (Ti) surfaces at days 1 and 7, respectively. Expression of the osteogenic marker genes was determined by real-time RT-PCR. Note that the expression levels are the highest on the Ln2-P3-coated surface both at day 1 and at day 7, as compared with the uncoated and SP-coated surfaces. Data are expressed as the mean ± standard deviation (*n* = 3). Ti, Ti + SP, and Ti + Ln2-P3 on the graphs mean the uncoated, SP-coated, and Ln2-P3-coated c.p. Ti surfaces, respectively.

The results of the alkaline phosphatase activity showed that the Ln2-P3-coated surface had a significantly higher ALP activity than the SP-coated and uncoated surfaces after 1 day of culture (*P* < 0.01; Figure 3(a)). Note that the enzyme activity on the SP-coated surface was significantly lower than that on the uncoated surface at this time point. By day 3, there was no significant difference in ALP activity among the various coated and uncoated surfaces (Figure 3(b)). Quantitative real-time PCR results also indicated that the Ln2-P3-coated surface displayed a significantly higher expression of both osteogenic markers (ALP and bone sialoprotein) at both day 1 and day 7, as compared with the SP-coated and uncoated surfaces (*P* < 0.01; Figures 4(a) and 4(b)).

## 4. Discussion

The Ln2-P3 peptide, the DLTIDDSYWYRI motif, has a positive effect on epithelial cell attachment [10–12]. Here, we found that the Ln2-P3 peptide enhanced bone cell attachment and the expression of osteoblast differentiation markers. The Ln2-P3 peptide may be a candidate for surface functionalization. Since the Ln2-P3 peptide consists of only 12 amino acids, it is expected to bypass an immune reaction, and in light of the present results, it is anticipated that this peptide will reduce the overall treatment time in dental implant therapy by promoting bone cell adhesion and enhancing osseointegration between the host bone and the implant surface.

The roughness of an inserted implant surface is known to have an effect on the bone response around the implant [9, 23–25], with previous reports indicating that an *S*_a_ value of about 1.5 *μ*m provides an optimal bone response [23, 25]. This 1.5 *μ*m value was, however, obtained using blasted surfaces only. The optimal roughness of anodized and Ca-P-coated surfaces has not been studied. In this study, we showed that the highest cell attachment occurred with an anodized surface, even though the mean *S*_a_ value of the surface was 0.68 *μ*m, which is much lower than the optimal value of 1.5 *μ*m. The morphology and chemical compositions of the implant surfaces also affect the bone response [9, 26]; therefore, further studies are required to determine the influence of such factors on cell affinity and attachment.

The c.p. Ti surface showed a significantly lower cell attachment as compared with the anodized Ti surface. However, coating with the Ln2-P3 peptide reduced this difference in attachment considerably, suggesting that the peptide coating may have more of an effect on cell affinity than the base surface. By comparison, the SP peptide did not enhance cell attachment, indicating the importance of the DLTIDDSYWYRI motif. In this study, we investigated the peptide-coated surfaces without the use of a blocking agent to eliminate cell binding to the base surface because peptide-coated implants in the clinic are inserted without a blocking agent. This means that both the peptide and surface of the implant can affect the response of the surrounding tissue. Future studies are needed to determine the effect of the peptide on cell attachment after blocking the surface of the substrate.

ALP expression is widely used as an early/intermediate marker of osteogenesis and sialoprotein expression is used as an intermediate/late marker [27–29]. An increase in ALP activity is often associated with osteoblastic differentiation [30]. The increased gene expression levels and ALP activity associated with the Ln2-P3 coating indicated that the functional Ln2-P3 peptide may strongly affect bone cell activity. This was further confirmed by the reduced gene expression and lower ALP activity on the SP-coated surfaces. In vivo studies are necessary to investigate the bone response around the Ln2-P3-coated implants.

## 5. Conclusions

The Ln2-P3-coated implant surface promotes bone cell adhesion. From a clinical standpoint, this suggests that the Ln2-P3 coating on dental implants may decrease the bone healing time during repair procedures. However, the results of this in vitro study are limited, and further in vivo studies are necessary to clarify the clinical usefulness of an Ln2-P3-coated implant.

## Figures and Tables

**Table 1:** The means and standard deviations of the compositions and roughness for the investigated Ti disc surfaces.

	c.p. Ti	SLA	Anodized	(Ca-P) coated
Compositions (wt %)				
Ti	104.0 (0.4)	96.1 (1.0)	52.7 (2.4)	61.8 (2.7)
O	4.1 (0.4)	3.5 (0.3)	41.7 (0.6)	37.2 (1.9)
Ca			5.9 (0.7)	14.0 (0.5)
P			1.9 (0.3)	2.2 (0.4)
Roughness parameters				
*S*_*a*_ (*μ*m)	0.65 (0.05)	1.72 (0.26)	0.68 (0.02)	1.74 (0.09)
*S*_dr_ (%)	22.2 (2.5)	102.3 (21.7)	44.1 (4.3)	90.6 (9.1)
